# A Study on the Reliability of an On-Site Oral Fluid Drug Test in a Recreational Context

**DOI:** 10.1155/2016/1234581

**Published:** 2016-08-17

**Authors:** Stefano Gentili, Renata Solimini, Roberta Tittarelli, Giulio Mannocchi, Francesco Paolo Busardò

**Affiliations:** ^1^Drug Abuse and Doping Unit, Department of Therapeutic Research and Medicines Evaluation, Istituto Superiore di Sanità, Rome, Italy; ^2^Unit of Forensic Toxicology (UoFT), Department of Anatomical, Histological, Forensic and Orthopedic Sciences, Sapienza University of Rome, Rome, Italy

## Abstract

The reliability of DrugWipe 5A on site test for principal drugs of abuse (cannabis, amphetamines, cocaine, and opiates) detection in oral fluid was assessed by comparing the on-site results with headspace solid-phase microextraction (HS-SPME) gas chromatography-mass spectrometry (GC-MS) analysis on samples extracted by the device collection pad. Oral fluid samples were collected at recreational settings (e.g., discos, pubs, and music bars) of Rome metropolitan area. Eighty-three club goers underwent the on-site drug screening test with one device. Independently from the result obtained, a second device was used just to collect another oral fluid sample subsequently extracted and analyzed in the laboratory following HS-SPME procedure, gas chromatographic separation by a capillary column, and MS detection by electron impact ionization. DrugWipe 5A on-site test showed 54 samples (65.1%) positive to one or more drugs of abuse, whereas 75 samples (90.4%) tested positive for one or more substances following GC-MS assay. Comparing the obtained results, the device showed sensitivity, specificity, and accuracy around 80% for amphetamines class. Sensitivity (67 and 50%) was obtained for cocaine and opiates, while both sensitivity and accuracy were unsuccessful (29 and 53%, resp.) for cannabis, underlying the limitation of the device for this latter drug class.

## 1. Introduction

There has been increasing interest regarding a variety of alternative biological matrices such as oral fluid, sweat, and hair in the last few years [1, 2]. Specifically, oral fluid shows several advantages in the on-site screening for drug use. The collection is noninvasive and easy to perform; it can be achieved in privacy, under close supervision, thereby reducing any opportunity of sample adulteration [3].

Furthermore, oral fluid reflects blood-drug concentrations due to the correlation between kinetics of several drugs in the blood and oral fluid, suggesting recent drug use.

Recent data have demonstrated an improvement in some on-site drug testing to disclose current consumption of illicit drugs. This significant progress in the sample collection and the improved accuracy of analysis have determined a certain success of on-site tests on oral fluid [4–9].

Although international literature suggests that the manufacturers overstate the capabilities of on-site testing devices to detect drugs in oral fluids, a number of new on-site testing devices have been constantly developed [10–16].

These devices are being used in many countries to perform on-site testing on oral fluid controls in Driving Under the Influence of Drugs (DUID) [5, 7, 10, 11, 13, 15] and several recent publications demonstrate that oral fluid screening devices are becoming more robust and reliable [12, 17–20].

In Italy, since August 2010, the law has considered oral fluid as an alternative biological specimen for the determination of DUID. Specifically, the devices can be used for rapid on-site testing as a first screening [21].

Among the developed devices, DrugWipe® is an immunochromatographic test strip, based on the Frontline urine test strip from Boehringer Mannheim (F. Hoffmann-La Roche, Basel, Switzerland) [22]. A pink colour in the test window indicates the presence of the analyte to which the test is specifically addressed and different devices are needed for the detection of each class of drugs of abuse. A recent version of this device, DrugWipe 5A, can simultaneously reveal the presence of cannabis, amphetamine, methamphetamine, ecstasy, cocaine, and opiates in oral fluid of consumers. In detail, the device is divided into two parts with two different collection pads: one for opiates and cocaine and the other for amphetamines and cannabis.

Here is reported our experience with application of DrugWipe 5A on-site oral fluid testing in recreational settings, subsequent oral fluid collection, and quantitative detection of opiates, cocaine, amphetamines, and cannabis during preventive actions carried out by a nongovernmental organization (NGO) against drug use in recreational settings (e.g., discos, pubs, and music bars) of Rome metropolitan area (Lazio, Italy).

The study's aim was to verify the reliability of DrugWipe 5A device for an on-site drug screening and whether a second device could be used as a simple collector for a subsequent confirmatory chromatographic-mass spectrometric assay. Specifically, easy and low-cost solvent-free headspace solid-phase microextraction and gas chromatography-mass spectrometry for drugs abuse (amphetamines, opiates, cocaine, and cannabinoids) in oral fluid directly collected by the device pad has been used. The method has been applied in real cases of 83 drivers stopped during roadside controls.

## 2. Experimental

### 2.1. Chemicals, Reagent, and Device

Codeine, morphine, 6-monoacetylmorphine (6-MAM), codeine-d_3_, morphine-d_3_, 6-monoacetylmorphine-d_3_, amphetamine (A), methamphetamine (MA), methylenedioxyamphetamine (MDA), methylenedioxymethamphetamine (MDMA), methylenedioxyethamphetamine (MDE),* N*-methyl-1-(1,3-benzodioxol-5-yl)-2-butanamine (MBDB), ketamine, methadone, cocaine, cocaethylene, Δ9-Tetrahydrocannabinol (THC), 3,4-methylenedioxypropylamphetamine (MDPA), cocaine-d_3_ (COC-d_3_), Δ8-Tetrahydrocannabinol (Δ8THC), N-Methyl-N-(trimethylsilyl)trifluoroacetamide (MSTFA), N,O-bis(trimethylsilyl)trifluoroacetamide (BSTFA), and trimethylchlorosilane (TMCS) were purchased from Sigma Aldrich (Milan, Italy).

Ultrapure water was obtained from a Milli-Q Unit (Millipore, Bedford, MA, USA). Acetic anhydride, sodium hydroxide (NaOH), hydrochloric acid (HCl), sodium chloride (NaCl), potassium carbonate (K_2_CO_3_), and acetone of analytical grade were purchased from Carlo Erba (Milan, Italy).

A solid-phase microextraction SPME Holder (manual) assembly with a replaceable extraction fibre coated with 100 *μ*m polydimethylsiloxane (PDMS) and a 110 VAC block heater purchased from Sigma-Aldrich were used. DrugWipe 5A devices were provided by Securetec (Brunnthal, Germany).

### 2.2. Subjects and Oral Fluid Testing with DrugWipe 5A

During preventive actions (January to March 2015) carried out by NGO in the five principal discos, pubs, and music bars of Roma metropolitan area, 83 young people were tested with the DrugWipe 5A oral fluid screening device obtained from Securetec (Brunnthal, Germany). The participants were informed on the purpose of sample collection, and they gave signed consent to the collection and subsequent anonymous analysis of their oral fluid.

NGO staff performed oral fluid screening tests by wiping the tongue of the drug users 5–10 times with the collection pad, as recommended by the manufacturing instructions. After the sampling, the collection pad was put into direct contact with the drug test strip. Drug test and validity results were visually read after 10 minutes. Two coloured lines, one in the upper control window and one in the lower test window, indicate a positive result. Cut-off values for different drug groups provided by the manufacturer were the following: amphetamines, 50 ng/mL; methamphetamines, 25 ng/mL; MDMA, 25 ng/mL; cocaine, 30 ng/mL; opiates, 10 ng/mL; and cannabis, 30 ng/mL.

Another oral fluid sample was collected using DrugWipe 5A and the two collection pads were mailed to the analytical laboratory and stored at ambient temperature without any preservative until HS-SPME-GC-MS analysis was performed (for a maximum of 14 days).

In both cases of positive and negative results to the first screening test on oral fluid by DrugWipe 5A, a second sample was collected for chromatographic analysis.

### 2.3. Calibration Standards

Stock solutions of each analyte (1 mg/mL) were combined and diluted with methanol to set working calibrator solutions (0.01, 0.02, 0.05, 0.10, 1.00, and 2.00 *μ*g/mL). Working internal standard methanol solutions at 2 *μ*g/mL were also prepared. Stock solutions were stored at −20°C until use. Blank oral fluid samples were obtained by wiping the tongue of laboratory staff 5–10 times with the DrugWipe 5A test pad.

Oral fluid calibrations were prepared by spiking 5 *μ*L of working calibrator solutions and internal standard solutions directly onto the test pad area of the blank sample.

Quality control samples of 0.075 ng/pad (low control), 0.15 ng/pad (medium control), and 0.45 ng/pad (high control) for THC and 0.45 ng/pad (low control), 2.00 ng/pad (medium control), and 4.00 ng/pad (high control) for other drugs of abuse were prepared in the same oral fluid drug-free pad and stored until analysis. They were included in each analytical batch to check calibration, accuracy and precision, and stability of samples under storage conditions.

Although recently some authors claim the volume to be about 20 *μ*L [23], other authors reported that limited or unknown collection volume from the collection device might create a number of difficulties for the laboratory [24, 25]. For this reason, the concentration of the analytes was expressed in ng substance/pad.

### 2.4. Oral Fluid Analysis

Oral fluid samples collected by a second DrugWipe 5A device were analyzed by headspace solid-phase microextraction (HS-SPME) and gas chromatography-mass spectrometry (GC-MS) procedures according to Merola et al. [26] and Moller et al. [27]. In particular, although ketamine was not among the substances screened by DrugWipe, HS-SPME-GC-MS investigated it for information on current drug consumption.

For the analysis of opiates, the first pad was removed and extracted with 200 *μ*L methanol in a closed headspace vial (2 mL), containing internal standard solution (5 *μ*L of 2 *μ*g/mL codeine-d_3_, morphine-d_3_, and 6-monoacetylmorphine-d_3_). The sample was incubated for 60 min at 60°C. The methanol extract was transferred to a 10 mL vial and dried under nitrogen flow, 10 *μ*L BSTFA + 1% TMCS were added, and the SPME needle was introduced into the vial and exposed to adsorption for 30 min at 125°C. Finally, thermal desorption of the fibre was performed at 250°C for 3 min inside the GC.

For cocaine, ketamine, and amphetamines, the second pad was removed and extracted with 200 *μ*L 1 M HCl in a closed headspace vial (20 mL), containing internal standards (5 *μ*L of 2 *μ*g/mL MDPA, COC-d_3_, MDPA, and Δ-8 THC). The sample was incubated for 60 min at 60°C.

After cooling at room temperature, the extracted acid layer was transferred to another vial (2 mL) containing 200 mg K_2_CO_3,_ the SPME needle was introduced, and the fibre was exposed to adsorption for 10 min at 90°C; 5 *μ*L acetic anhydride was added, and the SPME needle was introduced into a second vial and exposed for 3 min at 90°. Thermal desorption was performed at 250°C for 3 min inside the GC.

For THC extraction, 1 mL NaOH 1 M and 0.5 g NaCl were added to the vial containing the pad previously used; the SPME needle was introduced into the vial and exposed to adsorption for 30 min at 150°C. For derivatization, 5 *μ*L of MSTFA was added and the fibre was exposed for 10 min at 90°C. Thermal desorption was performed at 250°C for 3 min inside the GC.

### 2.5. GC-MS Analysis

A Gas Chromatography 6890 Plus and Mass Selective Detector 5973N (Hewlett-Packard, Palo Alto, CA, USA) equipped with a J&W 19091S-101 HP-5MS Trace Analysis capillary column (5% PH ME Siloxane; film thickness, 0.33 *μ*m; length, 12.5 m; column ID, 0.20 mm) was used. The column temperature was initially held at 60°C for 2 min and then raised 20°C/min to reach 250°C and finally held at 250°C for 5 min. The temperature of the injection port, ion source, and transfer line was set at 250°C, 230°C, and 280°C, respectively. Thermal desorption was performed at 250°C for 3 min inside the gas chromatograph. Helium was used as carrier gas at a flow rate of 0.7 mL/min. The splitless injection mode was used. The mass spectrometer uses electron impact ionization. The mass spectra were collected by total ion chromatography. Identification criteria were based on retention time (RT) ±0.02 min with respect to the samein spiked oral fluid sample and on the relative abundance of the three confirming ions with respect to the target. Quantitative data were obtained by selected ion monitoring for each compound and for internal standards (IS). Monitored ions and RT for each compound are shown in Table 1.

### 2.6. GC-MS Method Validation

Validation protocol applied in the present study included linearity, limit of detection (LOD), limit of quantification (LOQ), precision, accuracy, and stability as reported elsewhere [28–30].

Linearity was determined by least-squares regression with 1/*x* ^2^ weighting of the following concentration: 0.05, 0.1, 0.25, 0.50, and 1.00 ng/pad for THC and 0.25, 0.50, 1.00, 2.50, and 5.00 ng/pad for the other analytes. Acceptable linearity was achieved when the coefficient of determination was at least 0.99. The LOD and LOQ were evaluated with decreasing analyte concentrations in drug-spiked oral fluid samples. The LOD was defined as the lowest concentration with acceptable chromatography, the presence of all transitions with signal-to-noise ratios of at least 3, and a retention time within ±0.2 min of the average retention time of the calibrator. LOQ was the lowest concentration that met LOD criteria and a signal-to-noise ratio of at least 10.

Precision, accuracy, and analytical recovery were calculated from five different daily replicates for five different days of 0.075, 0.15, and 0.45 ng/pad for THC and 0.45, 2.00, and 4.00 ng/pad for other drugs of abuse.

Stability of analytes in the device pad was tested in triplicate at 0.50 ng/pad for THC and 5.00 ng/pad for other drugs of abuse left in the dark at room temperature for 7 and 14 days and then analyzed by HS-SPME-GC-MS.

### 2.7. Interpretation of the DrugWipe 5A Results

The evaluation of the results is based on classification into the following categories: true positive (TP), cases with a positive DrugWipe 5A test result and a positive HS-SPME-GC-MS analysis result; false positive (FP), cases with a positive DrugWipe 5A test result and a negative GC-MS analysis result; true negative (TN), cases with a negative DrugWipe 5A test result and a negative GC-MS analysis result; false negative (FN), cases with a negative DrugWipe 5A test result and a positive HS-SPME-GC-MS analysis result.

Taking into consideration the above classification, sensitivity, specificity, and accuracy of the DrugWipe 5A oral fluid were calculated as follows. Sensitivity = (TP/TP + FN*∗*100). Specificity = (TN/TN + FP*∗*100). Accuracy = (TP + TN/number  of  tests) [12, 15].

## 3. Results and Discussion

### 3.1. Validation Results

Linear calibration curves were obtained for the compounds under investigation with correlation coefficients (*R* ^2^) of at least 0.99 in all cases and LODs and LOQs values adequate for the purpose of the present study (Table 2).

Intraday and interday precision and accuracy of the analytes under investigation satisfactorily met the internationally established acceptance criteria and were always better than 15% (Table 3) and recovery ranged from 84.4% to 107.6% for the different compounds (Table 2).

With respect to stability test (Table 4), in samples stored in the dark, at room temperature, a maximum decrease of about 15% initial concentration was observed for amphetamines, cocaine, and opiates after 14 days. Conversely, in case of THC, a decrease of 50% initial concentration was already observed after seven days and it remained stable at the same percentage after fourteen days. This is in agreement with Crouch's study on the effects of the oral fluid collection device on THC concentration and on its stability [24]. For instance, apart from THC instability, the device pad resulted to be a reliable tool for oral fluid collection, which could be mailed and stored at ambient temperature for a maximum of 14 days.

### 3.2. Samples Analysis

In order to demonstrate the usefulness of the device DrugWipe 5A, the results obtained by the device were compared to the ones obtained by HS-SPME-GC-MS in eighty-three clubs goers. In case of HS-SPME-GC-MS, only substances detected by this assay were reported in Table 5.

The results of DrugWipe 5A analysis showed that 54 samples (65.1%) were positive to one or more substances: 8 were found to be positive to cannabis, 16 were found to be positive to amphetamines, and 8 were found to be positive to cocaine. Eight samples were positive to amphetamines and THC, 5 samples were positive to both amphetamines and cocaine, 2 samples were positive to THC and opiates, 3 samples were positive to cocaine and opiates, 1 sample was positive to amphetamines and opiates, and 3 samples were positive to amphetamines, cocaine, and opiates.

In the HS-SPME-GC-MS analysis, 75 samples (90.4%) were positive for one or more substances: 35 were polydrug users, 21 were positive only for THC (ranged concentration: <LOQ—11.82 ng/pad), 7 were positive for MDMA (ranged concentration: <LOQ—184.08 ng/pad), 8 were positive for cocaine (ranged concentration: <LOQ—1398.05 ng/pad), and 4 were positive for ketamine (ranged concentration: <LOQ—9.09 ng/pad), even if this substance was not included in DrugWipe screening.

DrugWipe 5A performance data (true positive, true negative, false positive, and false negative results) reported in Table 6 compared the device results with those obtained by HS-SPME-GC-MS and showed sensitivity, specificity, and accuracy of the device with respect to the different drug class.

The comparison between on-site and laboratory results confirmed the different reliability for each class of substances, as already reported in the literature [31].

From the obtained results, it can be said that the device performed quite well in detecting the amphetamines class, with sensitivity, specificity, and accuracy around 80% value.

The second best results were obtained in case of cocaine which showed good specificity and accuracy but worse sensitivity. In this concern, previous studies demonstrated that cocaine was predominantly found in oral fluid with respect to its principal metabolite benzoylecgonine, present in very low concentrations in this biological matrix [3, 9]. Furthermore, recently, it has been demonstrated that oral fluid concentration of benzoylecgonine and the relationship with cocaine are time dependent, unless cocaine is intravenously administered [32]. Since benzoylecgonine extraction by HS-SPME and detection would have presented a great analytical difficulty due to its polar nature, this metabolite was not considered in this study. Indeed, for the reported reasons, its detection would not have increased the number of samples positive to cocaine.

In case of opiates, an even poorer sensitivity value was calculated. The low prevalence for opiates in the studied group of drivers did not allow a proper evaluation of the performances of DrugWipe 5A for these substances.

Finally, in agreement with previous observations [13, 16], our results highlight the unsuccessful detection of THC by DrugWipe 5A device for oral fluid.

Observations by NGO staff and some laboratory test simulations have confirmed that the line test for cannabis is usually very weak and delayed [25]. This difficulty in interpreting the results may give rise to a high number of false negatives.

The comparison between device cut-offs with HS-SPME-GC-MS results confirms the high specificity (always >80%) for all class of substances and the poor sensitive value for opiates (about 67%) and THC (about 30%). On the other side, we observed an increase of sensitivity of both amphetamines (about 92%) and cocaine (about 80%).

Outside the principal aim of the study, our results evidenced a nonnegligible 20.5% of our clubs goers consuming ketamine. To the best of our knowledge, this is the first time the objective assessment of the consumption of this drug has been performed in oral fluid samples from a population of Italian disco goers. Significant limitation of on-site oral fluid test devices is that they cannot usually detect increasing number of new psychoactive drugs. Ketamine is only one of them. This is an issue that should be taken into account, especially when the device is applied at recreational settings, where the use of new drugs may be likely. It can be underlined that study limitation could lie in the fact that HS-SPME-GC-MS assay was not carried out in real oral fluid samples but precisely on extracts of collection pad. Nevertheless, this occurrence allowed a direct comparison of the immunochromatographic screening test and a confirmatory gas chromatographic-mass spectrometric assay on the same collected substrate. In addition, only eighty-three samples have been analyzed which cannot be conclusive but can be an eye opener on the reliability of this simple and easily applicable on-site test device for oral fluid drug testing.

## 4. Conclusions

From the results obtained in the present study, DrugWipe 5A device has been shown to be a practical, easy way of sampling and a non-time-consuming procedure for screening drug of abuse testing in oral fluid. The device has proven to be not sensitive and accurate enough for cannabis but acceptable for other drugs of abuse. Although oral fluid may be a useful matrix for on-site testing of drugged drivers, it is evident that it still shows a lack of sensitivity and, to ensure adequate reliability, GC-MS or LC-MS confirmation of on-site oral fluid screening tests remains necessary, due to the presence of a significant number of false negative and false positive results, even when using the commercial kit with the best performance.

## Figures and Tables

**Table 1:** Monitored ions and retention time (RT) for drugs of abuse in oral fluid samples by HS-SPME-GC-MS.

Compound	RT (min)	Ion *m*/*z* (relative abundance)
A	9.32	**86**, 91, 118
MA	9.37	**58**, 91, 100
MDA	11.32	**162**, 135, 221
Ketamine	11.36	**180**, 182, 209
MDMA	11.75	**58**, 100, 162
MDE	11.96	**72**, 114, 162
MBDB	12.07	**72**, 114, 176
MDPA (IS)^*∗∗*^	12.34	**86**, 128, 162
Methadone	13.03	**72**, 91, 294
COC-d_3_ (IS)^*∗∗*^	13.48	85, **185**, 306
Cocaine	13.50	82, **182**, 303
Cocaethylene	13.96	**196**, 272, 317
Δ8THC (IS)^*∗∗*^	14.52	303, 330, **386**
Δ9THC	14.71	303, 371, **386**
Codeine	16.95	178, 196, **371**
Codeine-d_3_ (IS)^*∗∗*^	16.99	181, 199, **374**
Morphine	17.70	236, 401, ** 429**
Morphine-d_3_ (IS)^*∗∗*^	17.73	239, 404, **432**
6-MAM	18.17	340, 357, ** 399**
MAM-d_3_ (IS)^*∗∗*^	18.19	343, 360, **402**

A, amphetamine; MA, methamphetamine; MDA, methylenedioxyamphetamine; MDMA, methylenedioxymethamphetamine; MDE, methylenedioxyethamphetamine; MBDB, *N*-methyl-1-(1,3-benzodioxol-5-yl)-2-butanamine; THC, Δ9-Tetrahydrocannabinol; MDPA, 3,4-methylenedioxypropylamphetamine; COC-d_3_, cocaine-d_3_; Δ8THC, Δ8-Tetrahydrocannabinol; 6-MAM, 6-monoacetylmorphine.

^*∗∗*^IS: internal standard.

Quantifier ions are in bold.

**Table 2:** Linearity of the HS-SPME-GC-MS procedure for compounds under investigation.

Compounds	Slope	Intercept	*R* ^2^	LOD (ng/pad)	LOQ (ng/pad)	Analytical recovery (%)
A	0.64	0.07	0.995	0.37	1.11	**100.8**
MA	2.08	0.20	0.990	0.68	2.04	**99.2**
MDA	1.93	−0.16	0.988	0.60	1.80	**88.8**
Ketamine	6.47	−1.16	0.990	0.73	2.19	**84.4**
MDMA	5.57	0.86	0.996	0.34	1.02	**100.8**
MDE	5.73	0.14	0.998	0.26	0.78	**103.2**
MBDB	8.34	0.77	0.999	0.11	0.33	**98.8**
Methadone	9.74	−1.46	0.990	0.71	2.13	**85.5**
Cocaine	6.18	0.05	0.996	0.35	1.05	**107.6**
Cocaethylene	2.01	−0.42	0.992	0.51	1.53	**90.4**
THC	10.52	0.03	0.990	0.06	0.18	**102.0**
Codeine	4.73	0.63	0.996	0.06	0.18	**96.0**
Morphine	7.32	2.05	0.995	0.43	1.29	**96.4**
6-MAM	9.52	3.74	0.993	0.39	1.17	**105.6**

A, amphetamine; MA, methamphetamine; MDA, methylenedioxyamphetamine; MDMA, methylenedioxymethamphetamine; MDE, methylenedioxyethamphetamine; MBDB, *N*-methyl-1-(1,3-benzodioxol-5-yl)-2-butanamine; THC, Δ9-Tetrahydrocannabinol; 6-MAM, 6-monoacetylmorphine.

**Table 3:** Intraday (*n* = 5) and interday (*n* = 15) precision (measured as coefficient of variation, CV%) and accuracy (measured as % error) for analytes under investigation in oral fluid samples.

Analyte	Intraday precision			Intraday accuracy			Interday precision			Interday accuracy		
	Low QC	Medium QC	High QC	Low QC	Medium QC	High QC	Low QC	Medium QC	High QC	Low QC	Medium QC	High QC
A	7.6	12.0	14.8	5.9	10.5	8.0	13.1	1.5	9.8	10.6	3.5	10.8
MA	14.8	3.1	11.7	6.4	9.3	7.4	10.2	7.8	4.4	10.4	9.7	8.0
MDA	5.3	8.2	5.9	6.2	10.3	11.6	9.6	2.4	8.8	10.5	8.5	9.4
Ketamine	9.2	7.1	9.5	4.3	11.1	10.3	8.5	7.4	10.1	10.1	8.8	10.3
MDMA	6.5	10.8	10.8	8.3	9.7	6.9	9.4	1.7	5.5	9.1	9.5	7.2
MDE	4.1	10.5	8.0	2.9	10.6	11.8	9.8	3.4	8.0	10.8	7.3	7.4
MBDB	10.6	5.1	9.4	3.4	10.4	10.7	11.5	12.3	7.4	12.2	10.6	10.2
Methadone	11.3	5.6	8.3	8.9	10.5	11.5	12.4	5.1	11.6	7.8	4.9	11.8
Cocaine	7.4	9.8	7.2	8.7	3.5	6.2	5.5	6.6	7.2	8.6	7.5	6.6
Cocaethylene	9.5	4.4	7.4	8.2	9.1	4.3	8.7	2.5	6.9	5.0	4.3	10.0
THC	11.8	8.8	10.2	5.9	10.8	8.0	10.2	1.6	11.8	4.8	5.2	8.7
Codeine	10.0	10.1	11.8	4.8	9.1	7.4	10.6	6.5	9.8	11.3	9.9	10.8
Morphine	6.5	7.8	10.1	5.3	7.8	11.6	13.1	3.5	4.4	6.8	3.5	10.8
6-MAM	6.2	8.0	10.0	1.9	8.6	9.8	10.2	7.4	8.8	9.9	9.7	8.0

A, amphetamine; MA, methamphetamine; MDA, 3,4-methylenedioxyamphetamine; MDMA, 3,4-methylenedioxymethamphetamine; MDEA, 3,4-methylenedioxy-N-ethylamphetamine; MBDB, N-methyl-1-(1,3-benzodioxol-5-yl)-2-butanamine; THC, Δ^9^-Tetrahydrocannabinol.

**Table 4:** Stability results at room temperature after 7-day and 14-day test.

Analyte	7-day test (%)	14-day test (%)
A	96.0	85.0
MA	95.2	84.8
MDA	95.0	85.3
Ketamine	97.9	88.9
MDMA	96.0	85.1
MDE	95.6	84.9
MBDB	95.9	84.9
Methadone	99.7	88.9
Cocaine	99.9	86.1
Cocaethylene	98.5	85.1
THC	51.0	50.0
Codeine	98.5	89.1
Morphine	98.0	89.0
6-MAM	97.9	88.7

A, amphetamine; MA, methamphetamine; MDA, 3,4-methylenedioxyamphetamine; MDMA, 3,4-methylenedioxymethamphetamine; MDEA, 3,4-methylenedioxy-N-ethylamphetamine; MBDB, N-methyl-1-(1,3-benzodioxol-5-yl)-2-butanamine; THC, Δ^9^-Tetrahydrocannabinol.

**Table 5:** DrugWipe 5A and HS-SPME-GC-MS results on oral fluid samples.

DrugWipe 5A						HS-SPME-GC-MS (ng/pad)						
	Sample	Cannabis	A	Cocaine	Opiates	THC	A	MDMA	Cocaine	Cocaethylene	Opiates	Ketamine
1	2310A											
2	2310B					0.22						
3	2310C											
4	2310D			+					<LOQ			
5	2310E			+					<LOQ			23.17
6	0611A											
7	0611B											
8	0611C											
9	0611D	+			+	0.51						
10	0611E	+	+						22.55			
11	0611F											
12	0611G					5.79						
13	1211A			+					5.13			
14	0312A		+			0.54		71.25				
15	0312B		+	+					54.83			
16	0312C		+			3.44		<LOQ				
17	0312D		+					184.08				
18	0312E		+			2.10		19.28				
19	0312F		+			0.44		15.23				
20	0312G		+			0.38		24.41				
21	1012A					<LOQ						
22	1012B					0.49						
23	1812A	+				11.82						
24	1812B											<LOQ
25	1812C											<LOQ
26	1812D			+					1398.05			
27	1812E			+	+	0.92		<LOQ	4.37			
28	1812F			+		3.17			<LOQ			
29	1812G		+					22.81				
30	1812H		+					18.60	2.30			
31	0101A			+	+	1.98		<LOQ			1.80 (methadone)	<LOQ
32	0101B		+	+		0.36		11.38	<LOQ			<LOQ
33	0101C		+	+				21.94				<LOQ
34	0101D		+	+				65.47	<LOQ			3.81
35	0101E			+		6.25		14.39	97.04	2.50		
36	0701A					1.52						
37	0701B		+	+	+				17.17	1.64		
38	0701C	+				0.82						
39	1401A		+					12.11				
40	1401B											9.09
41	1401C		+	+		0.45		17.60	1.80			71.65
42	1802A					1.43						
43	1802B			+		<LOQ			<LOQ			4.01
44	1802C					0.58						<LOQ
45	1802D	+				1.28						
46	1802E					0.37						
47	1802G					1.50						
48	0402A					0.58						
49	2502A		+		+	<LOQ	10.33	<LOQ				
50	2502D			+	+	<LOQ			<LOQ		15.08 (6-MAM)	9.71
51	2502E					<LOQ						8.73
52	2602B	+				4.85						<LOQ
53	1803A			+					1.68			
54	2603A	+				0.59						
55	2603B	+				1.43						
56	2005A		+			<LOQ					<LOQ (methadone)	<LOQ
57	2005B											
58	2005C					<LOQ						
59	2005D											<LOQ
60	2005E					<LOQ						
61	2005F		+			1.95		4.04				
62	1006A	+										
63	1006B	+	+			72.82		212.15				
64	1006C		+					4.16				<LOQ
65	1006D	+	+			0.38		<LOQ				
66	1006E	+				0.24						
67	1906W					1.29		<LOQ	<LOQ			
68	1906V							116.28				
69	1906Z					4.63						
70	2705A							<LOQ				
71	0904A							<LOQ				
72	2506C	+	+						<LOQ			
73	2506D		+					10,40	<LOQ		8.11 (methadone)	
74	2406B	+	+			0.52		<LOQ				
75	2406A	+			+	<LOQ			<LOQ			
76	2506A	+	+			1.12		<LOQ				
77	2506B	+	+			<LOQ		1.49				
78	2606A		+			<LOQ						
79	1806U		+			8.02		21.33	<LOQ			
80	1806Y		+			4.08		50.63	<LOQ			
81	1806A		+	+	+	5.61						
82	1806W	+	+					1.20				
83	1806Z		+	+	+			5.76	<LOQ	<LOQ		

A, amphetamine; MDMA, 3,4-methylenedioxymethamphetamine.

**Table 6:** Performance data of DrugWipe 5A in comparison with HS-SPME-GC-MS results.

	Cannabis	Amphetamines	Cocaine	Opiates
TP	14	26	16	2
FP	4	7	3	7
TN	30	43	56	72
FN	35	7	8	2

Number of tests	83	83	83	83

Sensitivity (%)	29	79	67	50
Specificity (%)	88	86	95	91
Accuracy (%)	53	83	87	89

TP, true positive; FP, false positive; TN, true negative; FN, false negative.
